# Frontal Analysis Continuous Capillary Electrophoresis Study on the Interaction of an Amphiphilic Alternating Copolymer with Triton X-100

**DOI:** 10.1155/2011/617981

**Published:** 2011-06-23

**Authors:** Akihito Hashidzume, Takuya Shimomachi, Takahiro Sato

**Affiliations:** Department of Macromolecular Science, Graduate School of Science, Osaka University, Toyonaka, Osaka 560-0043, Japan

## Abstract

The interaction of amphiphilic alternating copolymers of sodium maleate and dodecyl vinyl ether (Mal/C12) with a nonionic surfactant, Triton X-100 (TX), was investigated by frontal analysis continuous capillary electrophoresis (FACCE). The binding isotherms obtained from FACCE data were indicative of weak cooperative interaction for all the polymers examined. The cooperative interaction was also analyzed by the Hill model, and the results were compared with the previous results on the interaction of statistical copolymers of sodium 2-acrylamido-2-methylpropanesulfonate and *N*-dodecylmethacrylamide with TX.

## 1. Introduction

**Scheme 1:**
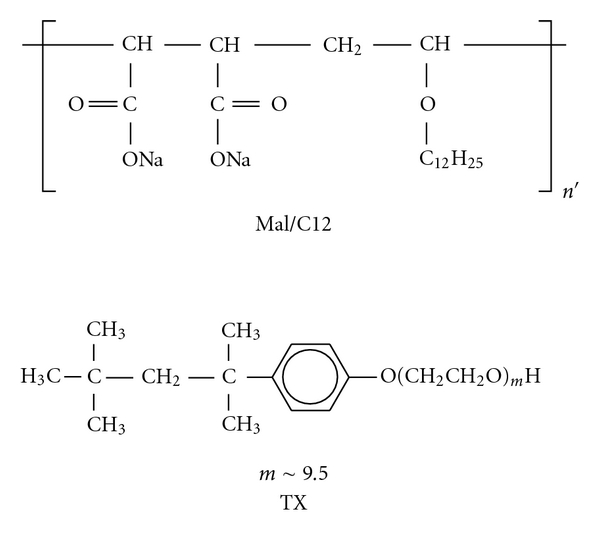
Chemical structures of Mal/C12 and TX.

Interactions between amphiphilic polymers and surfactants have attracted increasing interest from researchers in the last two or three decades because they are considered as simple model systems for colloid-colloid interactions, which are important in biological systems and various applications [1–7]. The interactions of amphiphilic polymers with surfactants have been investigated so far by various techniques [1], including equilibrium dialysis [8, 9], turbidimetry [10–12], viscometry [10, 13, 14], light scattering [10, 15], and fluorescence [10, 13, 15–17]. Frontal analysis continuous capillary electrophoresis (FACCE) is a powerful tool to investigate the association equilibrium of colloidal species because it allows ones to obtain binding isotherms, which are fundamental data for detail understanding on colloid-colloid interactions, in a short time period using a small amount of samples [18]. FACCE has been utilized mainly for binding equilibrium of protein-polymer systems [19–28]. To our knowledge, however, FACCE studies on the polymer-surfactant interaction have been still scarce [29, 30]. In the preceding study, the interaction of statistical copolymers of sodium 2-acrylamido-2-methylpropanesulfonate and *N*-dodecylmethacrylamide {A/C12(*x*), where *x* denotes the mol% content of *N*-dodecylmethacrylamide} with a nonionic surfactant, Triton X-100 (TX, Scheme 1), was investigated by FACCE [29]. The binding isotherms obtained using the FACCE data indicated that the binding of TX was weakly cooperative for the whole *x* range (*x *= 10–60 mol%) and A/C12(*x*) polymers of *x* > ca. 50 mol% exhibited higher cooperativity than did A/C12(*x*) copolymers of *x* < ca. 40 mol%. In this work, we have further studied on the interaction of an alternating copolymer of sodium maleate and dodecyl vinyl ether (Mal/C12, Scheme 1) with TX and compared the Mal/C12-TX interaction with the our previous study in order to investigate the effect of the polymer structure on the interaction with TX [29]. 

## 2. Material and Methods

The Mal/C12 polymers employed in this study were the same as those used in our previous studies [31–33]. These polymers were prepared by conventional free radical copolymerization of maleic anhydride and dodecyl vinyl ether using 2,2′-azobis(isobutyronitrile) as an initiator, followed by hydrolysis with NaOH [31–33]. Table 1 lists the characteristics of copolymers used in this study. *M*_*w*_ ranges (0.90–70) × 10^4^, and *M*_*w*_/*M*_*n*_ ranges 1.5–1.9. Triton X-100 (TX, Scheme 1) was purchased from Nakalai Tesque and used as received. Milli-Q water was used for all measurements. Other reagents were used without further purification.

A stock solution of 2 g/L Mal/C12 was prepared by dissolving each solid polymer sample (recovered by freeze-drying) in a 25 mM Borax (pH 9.3) with vigorous stirring for 15 min. A stock solution of 20 mM TX was prepared by dissolving TX in the same buffer. The stock solutions were stored overnight at room temperature for equilibration. Sample solutions for FACCE measurements were prepared by mixing the stock solutions of Mal/C12 and TX, and the borate buffer, fixing the polymer concentration (*C*_*p*_) at 1 g/L. All the sample solutions were equilibrated overnight and then filtered using a 0.2 *μ*m pore size disposable membrane filter prior to measurement. 

FACCE measurements were performed with a Beckman P/ACE 5510 instrument using a cartridge equipped with a bare fused silica capillary (Restek, i.d. = 50 *μ*m). The total length of the capillary was 27 cm, and the effective separation length (from inlet to detection window) was 20 cm. A 25 mM Borax (pH 9.3) was used as a run buffer. The capillary was conditioned by flushing 0.1 M NaOH and water successively at 20 psi for 1 min, followed by washing with the run buffer prior to use. After injection of a solution of neutral marker, mesityl oxide, for 2 sec, the capillary inlet end was transferred to a sample vial to initiate sample introduction and separation by applying a constant voltage of 10 kV at 25°C. The sample signal was detected by UV absorption at 214 nm. The details of FACCE instrumentation are described in the literature [18].

## 3. Results and Discussion

**Figure 1:**
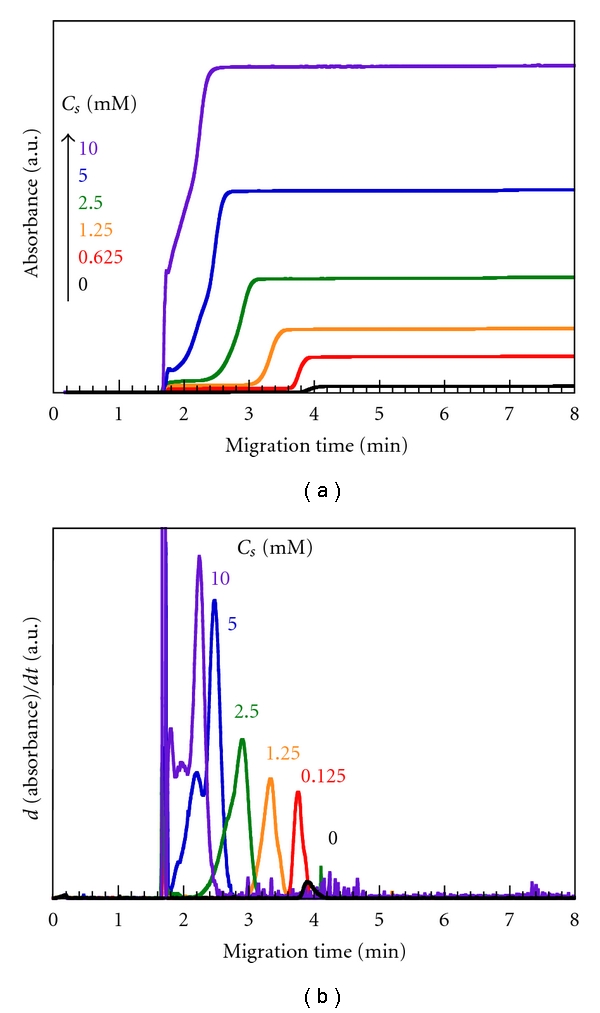
FACCE data (a) and migration time distributions (b) obtained by differentiation of the FACCE data for 1 g/L Mal/C12-2 in 25 mM Borax (pH = 9.3) in the presence of varying concentrations of TX.

Figure 1(a) shows examples of FACCE data for mixtures of 1 g/L Mal/C12-2 and varying concentrations of TX. The FACCE data in this figure were differentiated to obtain distributions of migration times, which correspond to conventional capillary electropherograms (Figure 1(b)) [34–36]. In Figure 1(b), signals at a migration time of ca. 1.8 min (=1.75 ± 0.07 min) are due to electrically neutral species, mesityl oxide (neutral marker), free TX micelles, and/or free TX unimers. In the absence of TX (i.e., *C*_s_ = 0 mM), the signal around 3.9 min is assigned to Mal/C12-2. In the presence of TX, the signals following the peaks for free TX are ascribable to complexes between the polymer and TX micelles. As *C*_s_ is increased, the migration time for the complexes decreases, indicating that the electrophoretic flow decreases. It has been reported that the electrophoretic flow of hydrophobically modified poly(sodium acrylate) decreases upon complexation with nonionic and zwitterionic surfactants because of an increase in the average friction of the monomers and bound surfactant micelles [37]. Thus, the decrease in the electrophoretic flow may be due to the increase in the average friction of the monomers and bound TX micelles. It should be noted here that, at *C*_*s*_ ≥ 5 mM, the signals due to the complexes are multimodal and considerably broad. These observations are indicative of broad distributions of size and composition in the complexes, that is, heterogeneous complexation. 

Electrophoretic mobility (*μ*) can be calculated as

(1) $$\mu =\frac{lL}{V}\left(\frac{1}{t_{s}}-\frac{1}{t_{0}}\right),$$

where *l* is the length of capillary between the anode and the detector, *L* is the total capillary length, *V* is the applied voltage, and *t*_*s*_ and *t*_0_ are the migration times for sample and the neutral marker, respectively [38]. For all the Mal/C12, the average values of *μ* ($\overset{̅}{\mu }$) were calculated using average values of *t*_*s*_ (${\overset{̅}{t}}_{s}$), which can be calculated as

(2) $${\overset{̅}{t}}_{s}=\frac{\int_{t_{1}}^{t_{2}}tA^{\prime }\left(t\right)dt}{\int_{t_{1}}^{t_{2}}A^{\prime }\left(t\right)dt}.$$

**Figure 2:**
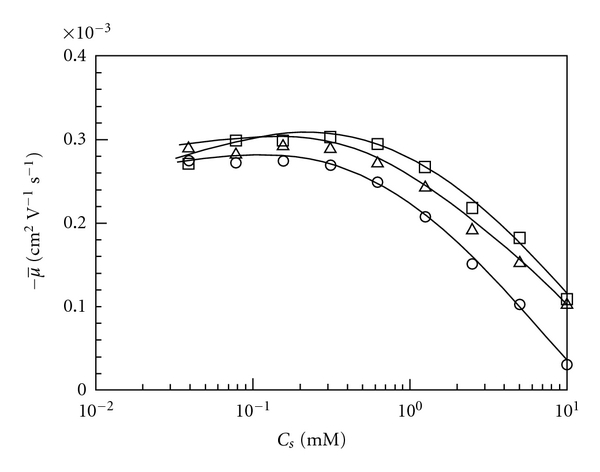
Electrophoretic mobility ($-\overset{̅}{\mu }$) as a function of *C*_*s*_ for mixtures of 1 g/L Mal/C12-1 (circle), Mal/C12-2 (square), and Mal/C12-3 (triangle) with TX in 25 mM Borax (pH = 9.3).

Here, *t* is the migration time, *A*′(*t*) is the differentiated FACCE electropherogram, and *t*_1_ and *t*_2_ are the migration times at which the signal due to the polymer-micelle complexes start and end in the differentiated FACCE electropherogram, respectively. It should be noted here that the ${\overset{̅}{t}}_{s}$ is apparent one not only because both Mal/C12 and TX are UV active but also because how the composition in the complex depends on migration time is unknown. Therefore, $\overset{̅}{\mu }$ is also apparent one. All the $\overset{̅}{\mu }$ values obtained are negative because the polymer-micelle complexes are negatively charged. Values of $-\overset{̅}{\mu }$ are thus plotted against *C*_*s*_ in Figure 2. It is noteworthy that $-\overset{̅}{\mu }$ values are similar for all the polymers examined at a *C*_*s*_, indicating that the electrophoretic mobility of Mal/C12-TX complexes is not strongly dependent on *M*_*w*_. The value of $-\overset{̅}{\mu }$ is almost constant at ca. 3 × 10^−4^ cm^2^ V^−1^ s^−1^ at *C*_*s*_ < 0.3 mM, but $-\overset{̅}{\mu }$ decreases from ca. 3 × 10^−4^ cm^2^ V^−1^ s^−1^ to ca. 1 × 10^−4^ cm^2^ V^−1^ s^−1^ with increasing *C*_*s*_ from ca. 0.3 mM to 10 mM presumably because of the increase in the average friction of the monomers and bound TX micelles [37]. 

**Figure 3:**
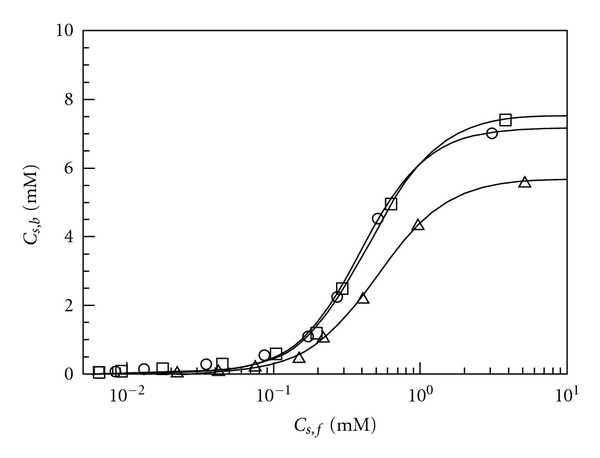
Binding isotherms for mixtures of 1 g/L Mal/C12-1 (circle), Mal/C12-2 (square), and Mal/C12-3 (triangle) with TX in 25 mM Borax (pH = 9.3). The best-fitted curves using the Hill model (3) are also drawn.

Using the signal intensities (absorbances) in the FACCE data (Figure 1(a)) and a calibration curve prepared from CE data for polymer-free TX, the total concentrations of TX molecules existing as free (i.e., unbound) micelles and free molecules (unimers) in the bulk phase (*C*_*s*,*f*_) and of TX molecules bound to Mal/C12 (*C*_*s*,*b*_) were calculated. The value of *C*_*s*,*f*_ was calculated from the abrupt increase in absorbance at ca. 1.7 min, which corresponded to the signal due to free TX unimers and micelles in the differentiated FACCE data. The value of *C*_*s*,*b*_ was calculated from the difference between the absorbance due to free TX unimers and micelles and that in the flat region at longer migration times by subtracting the absorbance due to Mal/C12 itself. For each data point, FACCE was measured three times, and the errors of *C*_*s*,*f*_ and *C*_*s*,*b*_ were confirmed to be less than 5%. The values of *C*_*s*,*b*_ are plotted as a function of *C*_*s*,*f*_ in Figure 3 to obtain binding isotherms. For all the polymers examined, binding isotherms are sigmoidal, indicative of cooperative binding of TX to Mal/C12. Since the onset of the cooperative binding is in fair agreement with the cmc of TX (*≈*0.2–0.4 mM) [39], it is likely that the cooperative binding is due to the formation of mixed micelles of the polymer with TX. 

In the case of cooperative binding of a small molecule to a polymer possessing a number of binding sites, such as the binding of an ionic surfactant molecule to an oppositely charged polyelectrolyte [40–44], the binding of the small molecule at one site increases the affinity for the molecule at adjacent sites. To account for such binding cooperativity, two common models are often applied [45, 46]: the Hill model [47] and the Zimm-Bragg model [48]. These models may not be apparently suitable to apply to the interaction of Mal/C12 with TX micelles because the cooperativity for this kind of systems is due to the formation of mixed micelles of amphiphilic polyelectrolytes with nonionic surfactants. However, we attempted to fit the Hill model to binding isotherms for the Mal/C12-TX system, because it is important to compare the present system with the previous work, that is, the system of statistical copolymers of sodium 2-acrylamido-2-methylpropanesulfonate and *N*-dodecylmethacrylamide {A/C12 (*x*), where *x* denotes the mol% content of *N*-dodecylmethacrylamide} with TX [29]. 

**Figure 4:**
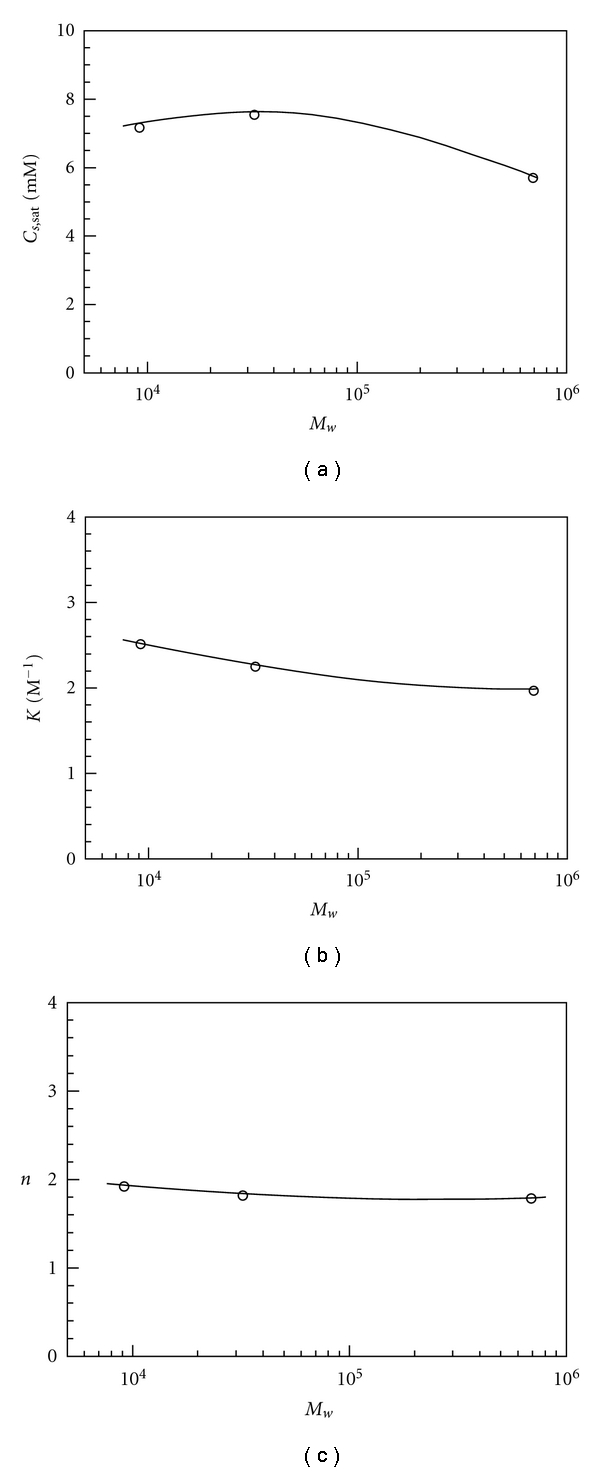
Parameters, *C*_*s*,sat _, *K*, and *n*, obtained by fitting using the Hill model (3) as a function of *M*_*w*_ for binding of TX to Mal/C12.

According to the Hill model, *C*_*s*,*b*_ is given as [47]

(3) $$C_{s,b}=C_{s,\mathrm{sat}\;}\frac{{\left(KC_{s,f}\right)}^{n}}{1+{\left(KC_{s,f}\right)}^{n}},$$

where *C*_*s*,sat _ is the concentration of bound TX at saturation *K* is the binding constant, and *n* is the Hill coefficient. The Hill coefficient, *n*, is a parameter for cooperativity, being unity for noncooperative binding (i.e., Langmuir-type binding) and larger than unity for cooperative binding. Figure 3 also includes the best-fitted curves using (3). For all the polymers, the curves fit in well with the experimental data. The parameters *C*_*s*,sat _, *K*, and *n* evaluated from the best fit are plotted against *M*_*w*_ as can be seen in Figure 4. All the panels of this figure indicate that the Mal/C12-TX interaction is not strongly dependent on *M*_*w*_, implying that Mal/C12 polymers have similar hydrophile-lipophile balances (HLB) based on the repeat unit. Figures 4(a) and 4(b) show that *C*_*s*,sat _ and *K* slightly decrease from ca. 7.4 mM and ca. 2.5 × 10^3^ M^−1^ to ca. 5.7 mM and ca. 2.0 × 10^3^ M^−1^, respectively, with increasing *M*_*w*_ from 9.0 × 10^3^ to 7.0 × 10^5^. Figure 4(c) indicates that *n* is practically constant (=1.8–1.9) independent of *M*_*w*_. On the basis of our previous study, it is considered that Mal/C12-1 and Mal/C12-2 form unicore flower-like micelles whereas Mal/C12-3 form multicore micelles depicted as flower-necklaces [33]. It is thus likely that the difference in the micellar structure is responsible for the slight decrease in *C*_*s*,sat _ and *K*. 

Here, these parameters for Mal/C12 are compared with those for A/C12(50), which has the same content of hydrophobic comonomer, in our previous study [29]. *C*_*s*,sat _, *K*, and *n* were determined to be 3.8 mM, 3.2 × 10^3^ M^−1^, and 3.4, respectively, for A/C12(50). FACCE measurements were carried out at 1 g/L for both the polymers, but the concentrations of dodecyl groups (*C*_C12_) are different because of different molar masses of the repeat unit. The ratios of *C*_*s*,sat _ to *C*_C12_ (*C*_*s*,sat _/*C*_C12_) were thus calculated to be 2.5(±0.4) and 1.8 for Mal/C12 and A/C12(50), respectively. These *C*_*s*,sat _/*C*_C12_ values indicate that a dodecyl group in Mal/C12 interacts with a larger number of TX molecules than does that in A/C12(50) at saturation, suggesting that intrapolymer hydrophobic interactions of dodecyl groups are stronger in the A/C12(50)-TX mixed micelles than in the Mal/C12-TX mixed micelles. *K* values { = (2.2 ± 0.3) × 10^3^  and  3.2 × 10^3^ M^−1^  for  Mal/C12  and  A/C12(50), resp.} demonstrate that A/C12(50) interacts more strongly with TX than does Mal/C12, indicating that polymer micelles of A/C12(50) are more hydrophobic than those of Mal/C12. Values of *n*{= (1.8 ± 0.1) and 3.4 for Mal/C12 and A/C12(50), resp.} show that the A/C12(50)-TX interaction is more cooperative than the Mal/C12-TX interaction although, in both cases, *n* values are not so large. These observations should be caused by the difference in the structure of the polymers. The remarkable difference is the numbers of charges per dodecyl hydrophobe: Mal/C12 has two charges per dodecyl hydrophobe whereas A/C12(50) has a charge per dodecyl hydrophobe. Therefore, the HLB of Mal/C12 is higher than that of A/C12(50), and A/C12(50) is more hydrophobic, resulting in the difference in the interaction behavior with TX. 

## 4. Conclusion

This paper described an FACCE study on the interaction of Mal/C12 with TX. Using FACCE data, the concentrations of free and bound TX were determined and binding isotherms were prepared. The binding isotherms were indicative of cooperative interaction for all the polymers examined. Analyzing the cooperative interaction by using the Hill model, *C*_*s*,sat _/*C*_C12_, *K*, and *n* were estimated to be 2.5 (±0.4), (2.2 ± 0.3) × 10^3^ M^−1^, and 1.8 ± 0.1 for Mal/C12. These values were slightly different from those for A/C12(50) (*C*_*s*,sat _/*C*_C12_ = 1.8, *K* = 3.2 × 10^3^ M^−1^, and *n* = 3.4). These differences should be caused by the difference in the structure of the polymers, for example, the number of charges per dodecyl hydrophobe. 

## Figures and Tables

**Table 1:** Characteristics of polymers used in this study.

Polymer code	*M*_*w*_/10^4^	*M*_*w*_/*M*_*n*_^[a]^
Mal/C12-1	0.86^[b] ^	1.5
Mal/C12-2	3.4^ [c] ^	1.8
Mal/C12-3	70^ [d]^	1.7

^[a] ^Determined by size exclusion chromatography for unhydrolyzed samples in tetrahydrofuran. Molecular weights were calibrated with polystyrene standards.

^[b] ^Calculated from the *M*_*n*_ determined by vapor pressure osmometry and the *M*_*w*_/*M*_*n*_ determined by size exclusion chromatography for an esterified sample.

^[c] ^Determined by sedimentation equilibrium in methanol containing 0.1 M LiClO_4_.

^[d]^Determined by static light scattering in methanol containing 0.1 M LiClO_4_.
